# Depression and opinion of dental students regarding the hybrid learning model during the COVID-19 pandemic

**DOI:** 10.1186/s40359-023-01157-8

**Published:** 2023-04-14

**Authors:** Marco Felipe Salas Orozco, Wendy Yesenia Escobar de González, Nuria Patiño Marín, Jesús Ramón Castillo Hernández, Juan Carlos Hernandez-Cabanillas, Ivan Olivares Acosta, Ricardo Martinez Rider, Miguel Angel Casillas Santana

**Affiliations:** 1 Doctorado en Ciencias Odontológicas, Facultad de Estomatología, Universidad Autónoma de San Luis Potosí, San Luis Potosí, MFSO C.P. 78290 Mexico; 2 Doctora en Cirugía Dental. Profesora de Cariología e Investigadora, Facultad de Odontología, Universidad de El Salvador, San Salvador, El Salvador; 3 Department of Clinical Research, Facultad de Estomatología, Universidad Autónoma de San Luis Potosí, San Luis Potosí, C.P. 78290 Mexico; 4 Facultad de Psicología, Universidad Autónoma de San Luis Potosí, San Luis Potosí, C.P. 78290 Mexico; 5 Maestría en Estomatología con Opción Terminal en Ortodoncia, Facultad de Estomatología, Benemérita Universidad Autónoma de Puebla, Puebla, C.P. 72410 Mexico

**Keywords:** COVID-19, Depression, Dental students, Pandemics, Prevalence, Epidemiology

## Abstract

**Background:**

The global spread of COVID-19 forced schools at all educational levels to close, which was repeated in more than 60 countries. In addition, the COVID-19 pandemic has affected the mental health of dental students world wide. This study hypothesizes that the prevalence of depression in dental students from El Salvador is higher than that reported in studies from Europe, Asia, and North America.

**Methods:**

This study was an online cross-sectional survey performed at the Faculty of Dentistry of the University of Salvador. The PHQ-9 questionnaire was applied to know the level of depression of the students, and a questionnaire focused on learning the opinion of the students on the hybrid teaching model adopted. Approximately 450 students participated in both questionnaires.

**Results:**

Regarding the levels of depression present in the students, 14% had minimal depression, 29% had medium depression, 23% had moderate depression and, 34% had severe depression. The students had an excellent opinion regarding the hybrid learning model.

**Conclusions:**

The prevalence of depression in dental students in El Salvador seems to be higher than that reported in studies in non-Latin American countries. Therefore, universities must generate care plans for mental health to avoid these harmful effects on students during future contingencies.

## Introduction

The disease that caused the most recent pandemic that affected the world was initially identified in December 2019 in Wuhan, China. This respiratory viral disease was named coronavirus disease 2019 (COVID-19). Approximately three months later, on March 11, 2020, COVID-19 was declared a global pandemic by the World Health Organization. Four months after the COVID-19 virus was identified, the first patient diagnosed with COVID-19 in El Salvador was confirmed on March 18, 2020 [1]. According to the global trends of the COVID-19 pandemic [2], experts estimated that 20% of the total population of El Salvador could contract the virus and require hospitalization. Likewise, between 4 and 9% of those infected would require care in intensive care units, which could cause the collapse of the country’s health services [3]. The COVID-19 pandemic has been active in El Salvador for approximately two and a half years (from January 3, 2020, to August 2, 2022). During this period, about 191,000 positive cases of COVID-19 have been reported, of which 4,200 incidents have caused death. The fight against the pandemic in El Salvador has consisted of administering approximately 11 million doses of vaccines [1].

The rapid spread of the COVID-19 pandemic worldwide meant that vulnerable populations had to be contained at home. Students are among these vulnerable populations, so classes must be suspended at many different educational levels worldwide. This suspension, in turn, interrupted the student’s study plans and activities for a long time. Therefore, online teaching had to be used to try to compensate for the deficiencies in education caused by COVID-19 [4].

However, some university courses, such as dentistry, require theoretical learning and constant clinical practice. Therefore, the clinical practice was the most challenging aspect to compensate for due to the high risk of transmission of COVID-19 and because dental schools had to suspend them entirely. At the same time, they developed strategies to allow students to return to clinical practice safely [5]. Dental education is based on three parts. The first part is the theory, which can easily be carried out through online classes. The second is practical training in simulation labs; virtual reality simulations; however not all faculties worldwide (especially in Latin America) have virtual reality simulators for this purpose. Finally, the third component is clinical practice, which can hardly be replaced. Therefore, it is vital to know the students’ opinions on the measures taken during the pandemic to implement hybrid learning models, especially in the Latin American context. In the final months of the pandemic, many dental schools opted for a hybrid education model. The hybrid learning model combines theoretical online teaching with clinical practices to carry out education safely. Mainly, in the final months of the pandemic, the administration of vaccines to the general populous kept the pandemic in control [6, 7].

Likewise, the prolonged confinement during the COVID-19 pandemic also caused a deterioration in the population’s mental health within the central psychological affections are stress, anxiety, and depression. In addition, it has been previously reported that catastrophic events (such as pandemics) that have economic and social consequences increase the prevalence of mental illnesses in the population [8]. This prevalence compounds students’ psychological problems due to the tremendous cognitive demand and economic issues they present during their university career development, even under normal conditions [9]. Many articles have been published on general depression during the COVID-19 pandemic. However, according to the literature, few studies have been carried out in populations of dental students, and even fewer meet quality criteria. Of these, only one has been carried out in a Latin American population such as Brazil. Therefore, we consider that this article contributes to the study of the prevalence of depression in Latin American dental students. The prevalence in this population can be very different from the others due to specific social and economic factors present in Latin America (for example, gender inequalities, lower economic income, less access to technology and less access to psychological care in Latin American countries) that are very different to those present in first world European, Asian or North American countries [10–12].

This study hypothesizes that the prevalence of depression in dental students from El Salvador is higher than that reported in studies from Europe, Asia, and North America. This study aims to know the different degrees of depression among dental students from the University of El Salvador Faculty of Dentistry and their opinion on the effectiveness of the hybrid model of learning implemented during the final months of the COVID-19 pandemic. Therefore, the first objective of this study is to know the different degrees of depression among dental students at the Faculty of Dentistry of the University of Salvador. The study’s second objective is to know the opinion on the effectiveness of the hybrid learning model that the University has implemented during the final months of the pandemic.

## Materials and methods

### Study type

This was an observational, descriptive, and analytical study. The questionnaires used in this study were distributed to dental students at the Faculty of Dentistry of the University of Salvador between October, November, and December 2021 (Tables 1 and 5). The questionnaires were applied individually through the google forms platform.

**Table 1:** Questionnaire to determine levels of depression of dental students (PHQ-9).

Over the last four weeks, how often have you been bothered by any of the following problems?					
		Not at all	Several days	More than half the days	Nearly every day
1. Little interest or pleasure in doing things?		0	1	2	3
2. Feeling down, depressed or hopeless		0	1	2	3
3. Trouble falling asleep, staying asleep, or sleeping too much		0	1	2	3
4. Feeling tired or having little energy		0	1	2	3
5. Poor appetite or overeating		0	1	2	3
6. Feeling bad about yourself - or that you’re a failure or have let yourself or your family down		0	1	2	3
7. Trouble concentrating on things, such as reading the newspaper or watching television		0	1	2	3
8. Moving or speaking so slowly that other people could have noticed. Or, the opposite - being so fidgety or restless that you have been moving around a lot more than usual		0	1	2	3
9. Thoughts that you would be better off dead or hurting yourself in some way		0	1	2	3

**Table 2:** Demographic characteristics from the questionnaire about the hybrid learning model

1. Age (median, range)	21 (17–36)
2. Gender	Frequency (%)
Male	116 (25)
Female	347 (75)
3. Semesters	Frequency (%)
II	105 (22)
IV	138 (30)
VI	82 (18)
VIII	25 (5)
X	31 (6)
XII	37 (9)
XIV	45 (10)

* The results are reported by frequencies and percentages

**Table 3:** Association between levels of depression with age group, gender, and semester of dental students

Factors	No depression	With depression	Total	Chi-square	p-Value
Age groups					
< 21	121	150	271	1,053	0.3048
> 21	95	97	192		
Gender					
Female	151	196	347	5,474	0.0193^a^
Male	65	51	116		
Semesters					
Initial	147	178	325	0,8853	0.3468
Advanced	69	69	138		

^a^p < 0.05 indicates a significant association with depression.

**Table 4:** Demographic characteristics from the questionnaire about the hybrid learning model

1. Age (median, range)	21 (17–36)
2. Gender	Frequency (%)
Male	105 (23)
Female	347 (77)
3. Semesters	Frequency (%)
II	101 (22.3)
IV	127 (28)
VI	75 (16.6)
VIII	32 (7)
X	39 (8.6)
XII	35 (8)
XIV	43 (9.5)

* The results are reported by frequencies and percentages

**Table 5:** Questionnaire to evaluate the attitude of students towards the hybrid learning model

Questions	Answers	Frequency	%	95% confidence interval
4. I am satisfied with the effectiveness of learning the online courses:	Strongly agree	29	6.4	0.044–0.091
	In agreement	90	19.9	0.163–0.239
	Neutral	227	50.2	0.455–0.549
	In disagreement	83	18.4	0.149–0.223
	Strongly disagree	23	5.1	0.033–0.076
5. The learning effectiveness of online courses is better than that of face-to-face courses:	Strongly agree	14	3.1	0.017–0.052
	In agreement	22	4.9	0.031–0.073
	Neutral	135	29.9	0.257–0.343
	In disagreement	157	34.7	0.303–0.393
	Strongly disagree	124	27.4	0.234–0.318
6. I think that professional dental lab format courses could change to online courses:	Strongly agree	18	4.0	0.024–0.063
	In agreement	35	7.7	0.055–0.107
	Neutral	97	21.5	0.178–0.255
	In disagreement	156	34.5	0.301–0.391
	Strongly disagree	146	32.3	0.280–0.368
7. Are you worried that covid-19 will create financial pressure for your school studies?	Strongly agree	144	31.9	0.276–0.364
	In agreement	161	35.6	0.312–0.402
	Neutral	114	25.2	0.213–0.295
	In disagreement	28	6.2	0.042–0.089
	Strongly disagree	5	1.1	0.004–0.027
8. Are you worried that the pandemic will affect your learning?	Strongly agree	236	52.2	0.474–0.568
	In agreement	139	30.8	0.265–0.352
	Neutral	66	14.6	0.115–0.182
	In disagreement	8	1.8	0.008–0.035
	Strongly disagree	3	0.7	0.001–0.020
9. My institution quickly adapted to hybrid learning:	Strongly agree	48	10.6	0.080–0.139
	In agreement	147	32.5	0.282–0.370
	Neutral	191	42.3	0.376–0.469
	In disagreement	48	10.6	0.080–0.139
	Strongly disagree	29	6.4	0.044–0.091
10. My institution has organized e-learning appropriately:	Strongly agree	44	9.7	0.072–0.129
	In agreement	150	33.2	0.288–0.377
	Neutral	179	39.6	0.350–0.442
	In disagreement	56	12.4	0.095–0.158
	Strongly disagree	23	5.1	0.033–0.076
11. My institution has provided students with training on teaching tools and software used for distance learning:	Strongly agree	37	8.2	0.059–0.112
	In agreement	131	29.0	0.248–0.334
	Neutral	165	36.5	0.320–0.411
	In disagreement	93	20.6	0.170–0.246
	Strongly disagree	26	5.8	0.038–0.084
12. For the online classes, I mainly used the following equipment:	Laptop/desktop pc	356	78.8	0.746–0.823
	Smartphone	90	19.9	0.163–0.239
	Tablet	5	1.1	0.004–0.027
	Computers in an institution outside of the University (for example, public library, internet cafe)	1	0.2	0.000–0.014
13. For the online classes, I mainly used the following network:	Own network	381	84.3	0.805–0.874
	Mobile data	68	15.0	0.119–0.187
	Public access point	1	0.2	0.000–0.014
	Network in an institution outside of the University (for example, public library, internet cafe)	2	0.4	0.000–0.017
14. The instructions given by most teachers (exam modes, task solving, etc.) are adapted to distance learning:	Strongly agree	39	8.6	0.062–0.117
	In agreement	150	33.2	0.288–0.377
	Neutral	185	40.9	0.363–0.456
	In disagreement	54	11.9	0.091–0.153
	Strongly disagree	24	5.3	0.035–0.079
15. Most teachers are making an effort to facilitate distance learning:	Strongly agree	88	19.5	0.159–0.234
	In agreement	191	42.3	0.376–0.469
	Neutral	125	27.7	0.236–0.320
	In disagreement	36	8.0	0.057–0.109
	Strongly disagree	12	2.7	0.014–0.047
16. Generally, the teaching materials are adequate for the technical demands of online learning:	Strongly agree	31	6.9	0.047–0.096
	In agreement	164	36.3	0.318–0.409
	Neutral	188	41.6	0.370–0.463
	In disagreement	56	12.4	0.095–0.158
	Strongly disagree	13	2.9	0.016–0.049
17. Teachers have generally organized and adapted to online learning:	Strongly agree	57	12.6	0.097–0.161
	In agreement	170	37.6	0.331–0.422
	Neutral	176	38.9	0.344–0.436
	In disagreement	38	8.4	0.060–0.114
	Strongly disagree	11	2.4	0.012–0.044
18. Which of the following was the most used methodology to teach?	Online classes in zoom	63	13.9	0.109–0.175
	Online classes in teams	24	5.3	0.035–0.079
	Online classes in google classroom	171	37.8	0.333–0.424
	Online classes on the university platform	181	40.0	0.355–0.447
	Whatsapp groups	2	0.4	0.000–0.017
	Daily or weekly tasks	11	2.4	0.012–0.044
19. I am concerned about the lack of practical education:	Strongly agree	280	61.9	0.572–0.664
	In agreement	125	27.7	0.236–0.320
	Neutral	44	9.7	0.072–0.129
	In disagreement	1	0.2	0.000–0.014
	Strongly disagree	2	0.4	0.000–0.017
20. I am afraid that it will not be possible to make up for the lack of practical education during my studies:	Strongly agree	171	37.8	0.333–0.424
	In agreement	149	33.0	0.286–0.375
	Neutral	104	23.0	0.192–0.272
	In disagreement	23	5.1	0.033–0.076
	Strongly disagree	5	1.1	0.004–0.027
21. I feel safe with the measures adopted by my institution to continue with clinical and laboratory practice:	Strongly agree	74	16.4	0.131–0.201
	In agreement	140	31.0	0.267–0.354
	Neutral	169	37.4	0.329–0.420
	In disagreement	43	9.5	0.070–0.126
	Strongly disagree	26	5.8	0.038–0.084
22. I feel confident in serving patients in clinical practices:	Strongly agree	47	10.4	0.078–0.136
	In agreement	95	21.0	0.174–0.251
	Neutral	167	36.9	0.325–0.416
	In disagreement	90	19.9	0.163–0.239
	Strongly disagree	53	11.7	0.089–0.151
23. The pandemic has affected my manual dexterity, and this is reflected in the quality of the treatments I perform:	Strongly agree	74	16.4	0.131–0.201
	In agreement	118	26.1	0.221–0.304
	Neutral	196	43.4	0.387–0.480
	In disagreement	49	10.8	0.082–0.141
	Strongly disagree	15	3.3	0.019–0.055
24. I feel confident caring for patients who have recovered from COVID-19:	Strongly agree	71	15.7	0.125–0.194
	In agreement	123	27.2	0.232–0.316
	Neutral	175	38.7	0.342–0.433
	In disagreement	55	12.2	0.093–0.156
	Strongly disagree	28	6.2	0.042–0.089

* The results are reported by frequencies and percentages

### Inclusion and exclusion criteria

Inclusion criteria were enrolled students, students of both genders, and students of any age. The exclusion criteria were: students dropped out during the period of application of the questionnaires.

### Ethical approval

The approval of the ethics committee of the University of El Salvador was obtained.

### Questionnaire to determine levels of depression of dental students

The questionnaire used to determine the levels of depression of dental students was the Patient Health Questionnaire-9 (PHQ-9). This questionnaire consisted of two parts: the first included nine questions, and the second included a single question [13]. The PHQ-9 is a questionnaire that evaluates the presence of depression symptoms in the last four weeks. The questionnaire classified the symptoms into 4 degrees of depression, which were:

• Minimal/no depression (score: 0–4).
• Mild depression (score: 5–9).
• Moderate depression (score:10–14).
• Severe depression (score: 15–27).

In a recent study, the PHQ-9 showed good sensitivity (0.88), specificity (0.85), and 95% confidence interval (0.82 to 0.88). This study employed the Spanish version of the PHQ-9 questionnaire. The Spanish version previously reported a specificity of 88%, a sensitivity of 87%, and an accuracy of 88% [14]. The cut-off point used to determine clinically essential levels of depression (moderate to severe depression) was a value equal to or greater than 10 points [15, 16]. In addition, the questionnaire included three questions about the primary demographic data of the participants. Those three questions were about age, gender, and the year of the degree that the participant is studying [17] (Table 1).

### Questionnaire to evaluate the opinion of students towards the hybrid learning model

The questionnaire to evaluate the students’ opinions towards hybrid learning consisted of 24 questions. The questionnaire was developed based on questions asked in previous studies that have already been published and validated [18–20]. The wording of the questions reported in Table 5 was an English translation from the Spanish version. The first three questions were about the primary demographic data of the participants (Table 3). Questions 4–8 were about the effectiveness of online classes and factors that can affect student performance. The following three questions were about the mechanisms applied by the University to carry out online learning. Questions 12 and 13 were about the students’ tools to access online classes. Questions 14–17 were about the performance of professors during online courses. Questions 18 through 24 were about students’ clinical practice during the pandemic (Table 4).

### Statistical Analyses

The Netquest (GfK group, Núremberg, Germany) online application was used to obtain the study’s sample size. A population of 463 students, a heterogeneity of 50%, and a confidence level of 95% were used to calculate the minimum sample size required. The minimum sample size was 211 students. The data analysis was carried out with the software GraphPad Prism version 8.3.1. (Graph Pad Software Inc, California, USA). To obtain the level of depression of each student surveyed, we added the score of each question to get the total points. Finally, the levels of depression were divided into two categories, no depression (below 10) and depression (10 and above), by taking a recommended cut-off score of 10 [21] according to the cut-off point with a score of 10, determined in a previous study. Likewise, the different semesters reported by the students were grouped into two categories, beginning semesters (from semesters 2 to 8) and advanced semesters (from semesters 10 to 14). In both questionnaires, ages were reported as medians and ranges, and gender and semester studied were reported as frequencies and percentages. The analysis of factors associated with depression was performed using the Chi-square test. Cronbach’s alpha was calculated for the 21 questions that comprise the questionnaire to assess the students’ opinion on the hybrid learning model and the nine questions of the PHQ-9 questionnaire. The study used Cronbach’s alpha calculation in RStudio version 2021.09.1 + 372 “Ghost Orchid” Release (RStudio Team (2021). RStudio: Integrated Development Environment for R. RStudio, PBC, Boston, MA URL http://www.rstudio.com/.) and used the “alpha ()” function from the “psych” package.

## Results

### Sample Characteristics

The total number of students who answered the mental health survey was 463. 75% of respondents were women, and 25% were men. The median age of the participants was 21 years, with a range of 17 to 36 years of age (Table 2). The total number of dentistry students who answered the questionnaire on the effectiveness of the hybrid learning model was 452. 23% were men, and 77% were women. The median age of the participants was 21 years, with a range of 17 to 36 years of age (Table 4).

### Depression levels of dentistry students in El Salvador

**Fig. 1:**
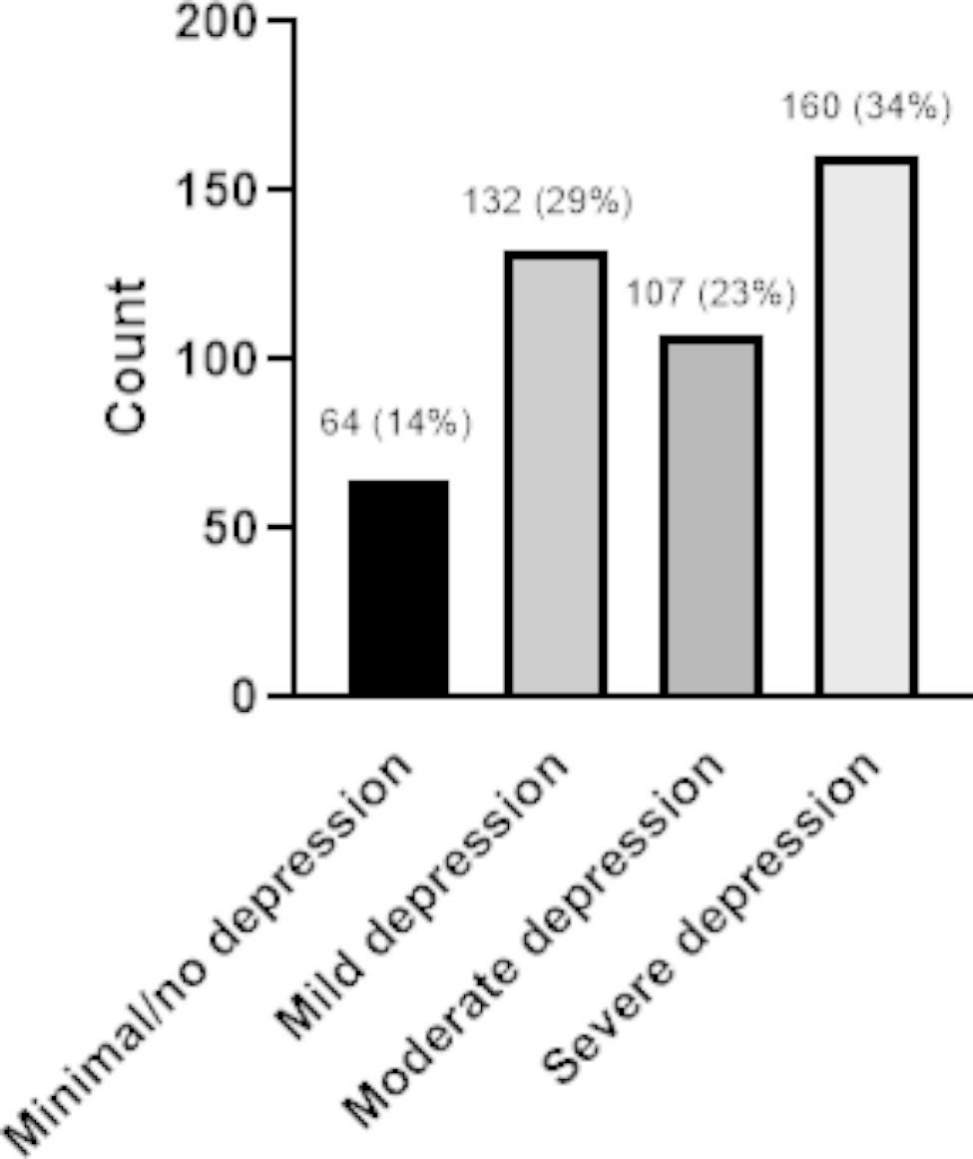
Results of the frequency and percentage of the different degrees of depression

*Cr*onbach’s alpha value for the PHQ-9 questionnaire was 0.86, with a 95% confidence interval of 0.76 to 0.92. According to the methodology of the PHQ-9 questionnaire, surveyed students’ levels of depression were classified into four groups, shown in Fig. 1. The entire study population answered the questionnaire (463 students). 43% of the students reported severe depression, 23% of the students reported moderate depression, and 29% of the students had medium depression. Finally, only 14% of the students did not present depression, or it was minimal (Fig. 1). Regarding the association of the variables of age group (< 21,>21), gender, and semester studied (initial or advanced) with the different levels of depression, only gender showed a significant association.

### Hybrid Learning Model Assessment

Regarding the questionnaire on the attitude and effectiveness of the hybrid model during the pandemic, Cronbach’s alpha value was 0.74 (acceptable) with a 95% confidence interval of 0.64–0.82.

#### Effectiveness of online classes and factors that can affect student performance (Questions 4–8)

Most students were neutral about the effectiveness of online learning, followed by disagreement with online learning (about 60%). Likewise, most students disagreed that the clinical practice laboratories could be taken online (about 67%), and most agreed that the epidemic would affect their learning and cause economic problems (about 66%).

#### Mechanisms applied by the University to carry out online learning (Questions 9–11)

42% of students considered that the University quickly adapted to the hybrid model, and the other 42% had a neutral opinion (question 9). Likewise, approximately 43% of students considered that the University organized online learning adequately, while the other 40% had a neutral opinion (question 10). Finally, 37% of the students considered that the institution provided adequate tools and training for online learning, while 36.5% had a neutral opinion (question 11).

#### Students’ devices to access online classes (Questions 12 and 13)

Around 80% of the students had their own laptop/desktop pc and internet network.

#### Professors’ performance during online classes (Questions 14–17)

Approximately 40% of the students agreed that the instructions given by the professors were well adapted to distance learning, while the other 60% had a neutral opinion (question 14). 60% of the students considered that the professors made an effort to facilitate distance learning for their students (question 15). Forty-three-point-2% of the students felt that the teaching materials during online learning were adequate, and about 40% had a neutral opinion (question 16). Approximately 40% of the students consider that the teachers have adapted to distance learning, while the other 60% had a neutral opinion (question 17).

#### Platforms used for online learning (question 18)

The three leading platforms used for online learning were the university platform, google classroom, and zoom.

#### Students’ clinical practice during the pandemic (Questions 19–24)

90% of students were concerned about the lack of clinical practice (question 19). 70% of the students considered that they could not recover the clinical and laboratory course during the rest of their studies (question 20). Around 40% of the students feel safe with the measures taken by the University to continue with clinical practices and laboratories, while the other 60% had a neutral opinion (question 21). Only 30% of students felt safe when treating patients, 37% had an impartial idea, and the remaining 23% did not feel safe (question 22). Approximately 53% of the students did not consider that they had lost manual dexterity during the pandemic (question 23). Finally, about 42% of the students felt safe when treating patients who recovered from COVID-19, while approximately 40% had a neutral opinion (question 24).

## Discussion

The study’s objectives were to know the degrees of depression of dental students during the contingency due to the COVID-19 pandemic and dental students’ opinions of hybrid learning implemented by the University of El Salvador Faculty of Dentistry during the COVID-19 pandemic. As far as we know, no studies have been carried out in Latin American countries where the presence of depression in dental students was investigated and where the opinion of students regarding the hybrid learning model was analyzed.

The PHQ-9 was the questionnaire used in this study to detect levels of depression in dental students [22]. This questionnaire has been widely used in previous similar studies [22, 23]. Other widely used questionnaires for the same purpose are the DASS-21 and HADS questionnaire [10, 24]. The total number of students who answered the PHQ-9 questionnaire was 463. Our study has the second largest sample size, only after the survey by Siddiqui & Qian (2021), in which the sample size was 655 students. Likewise, this study has the first place in sample size (463 students) in a Latin American country [25]. The second place is occupied by the study by Medeiros et al. in Brazil, with a sample size of 113 students [10].

The median age of the students who answered the two questionnaires was 21. This data coincides with similar studies in which a mean age of 21 was reported [10]. However, there are studies where the average age reaches 25 [24]. In this study, the percentage of women and men was 75% and 25%, respectively. These percentages are similar to those reported by previous studies. For example, in the survey by Medeiros et al., the authors noted that of the sample studied, 77% were women and, 23% were men [10]. In the German study by Mekhemar et al., the authors reported a percentage of women of 74% and men of 26% [26]. Two studies conducted in Malaysia reported 79% women and 21% men [25, 27]. Shailaja et al. reported 82% of women and 18% of men [28]. On the other hand, Hakami et al. reported more balanced percentages of men and women. The authors reported 55% women and 45% men [29]. The differences in the average age and the ratios of men and women between this study and previous studies are mainly due to the different populations studied. The differences in the number of respondents between the two questionnaires are because the questionnaires were applied independently.

Before the COVID-19 pandemic, depression in dental and medical students was approximately 28% in the US [30, 31]. Previous studies on the prevalence of depression during the general population pandemic report range from 12 to 31% [32, 33]. Deep et al. surveyed the pandemic in which they reported a 9% prevalence of depression in 199 dental students; in this study, the authors used the PHQ-9 questionnaire [34]. Knipe et al. also used the PHQ-9 questionnaire during the pandemic to report the prevalence of depression in dental students. The authors found a prevalence of depression of 35.4% in 344 dental students [35].

This study’s prevalence of moderate and severe depression (> 10) was 57%. The increased prevalence of depression may be due to the COVID-19 pandemic, which exerts more psychological stress on dental students than they experience under normal conditions. This percentage coincides with similar studies also carried out during the covid-19 pandemic. For example, Medeiros et al. reported with the PHQ-9 a prevalence of depression of 39.4% in 113 dental students in Brazil during the COVID-19 pandemic [10]. Chi et al. also registered with the PHQ-9 a prevalence of depression of 14.4% in 14 US dental students. However, the author’s sample size was meager, invalidating the results [23]. Kwaik et al. reported a 70% prevalence of depression in 305 Palestinian dental students. However, the questionnaire used for screening for depression was not the PHQ-9; the authors used the DASS-21 questionnaire, which could explain the high percentage of depression reported [36]. Hakami et al. used the DASS-21 questionnaire to register a prevalence of depression of 60.7% in 422 Saudi Arabian students [29]. Gas et al. used the DASS-21 questionnaire to report a prevalence of depression of 27.2% in 190 dental students from Turkey [37]. It is crucial to consider that the studies mentioned above were carried out during the initial and intermediate stages of the development of the pandemic. In contrast, our research was carried out in the final step. This difference in methodology could explain the considerable variation in the reported percentages of depression questionnaires used to detect depression and the different sample sizes. Finally, our study found a positive association between the degree of depression and female gender, coinciding with the report by Medeiros et al. [10]. However, other studies do not find an association between gender and levels of depression [25]. In general, this study’s prevalence of depression in dental students (57%) is higher than that reported in previous studies in Europe, Asia, and North America. For example, in a study that analyzed the mental health of medical science students (including dental students) in 9 countries, an overall prevalence of depression of 40% was found. This study included the countries of Mexico, Colombia, Venezuela, Chile, Brazil, Spain, Germany, Italy, and Japan [38].

A study in the USA reported a prevalence of depression of 14.4% [23]. Two studies conducted in India registered a prevalence of depression of 53.5% and 20% [22, 28]. Alfadley et al. reported a prevalence of depression of 10.9% [24]. Likewise, two studies in Malaysia reported depression in dental students at 24% and 33.6% [25, 27]. In addition, some studies report that COVID-19 infection in relatives of dental students multiplies by three the probability that they will develop symptoms of depression [39]. The above analyses were conducted during the COVID-19 pandemic and in dental students.

Regarding dental students’ attitudes towards the hybrid learning, questions 4 through 8 assess the effectiveness of online classes. Most dental students were neutral (50%) or disagreed (55%) on the efficacy of online learning, which coincides with similar studies reporting that 45% of dental students surveyed indicate that online learning needs to improve to be more effective [19]. In questions 9, 10, and 11 were about the mechanisms applied by the University to carry out online learning, 40% of the students had a neutral opinion, and another 40% agreed that the faculty had adequately adapted to the hybrid model and provided the appropriate tools for online learning. In a study in Jordan, students reported feeling comfortable (54%) with how the faculty implemented online teaching [20]. So, the hybrid model applied in the faculty of El Salvador has a degree of acceptance similar to those used in other parts of the world. Likewise, in this study (questions 12 and 13), 80% of the students had the necessary tools to take classes online. Access to online courses is similar to other studies; for example, in a survey conducted in India, 86.1% of students reported accessing online classes regularly [40]. In questions 14–17 (professors’ performance during online courses), 40 and 60% of the students consider that the teachers adapted excellently to online teaching. A similar study affirms this data in Italy, where dental students indicated that 70% of teachers had successfully adapted to online instruction [41]. In this study, the most used platforms to take classes online were the university platform, google classroom, and zoom. These data are very similar to a study in Brazil, where the leading platforms were virtual meetings (Zoom/Skype), the educational platform Moodle and the University system [42]. In questions 19–24 (students’ clinical practice during the pandemic), 90% of dental students are concerned about the lack of clinical practice. Several similar studies during the COVID-19 pandemic are consistent with these findings. For example, Etajuri et al. report that more than 50% of dental students do not feel satisfied with the clinical practice received during the pandemic [43]. Hattar et al. said 87% of dental students indicated their clinical practices were affected during the pandemic [20]. Finally, in this study, less than half of the students reported feeling safe when treating patients or with the protection measures adopted by the faculty. This trend has been reported in previous studies [44]. The general result of the questionnaire on the hybrid learning model indicates that the students were not affected by this learning model, which seems to contradict the depression levels obtained in this study and the results of similar studies. For example, a study conducted at a Lebanese University reported that online learning is associated with increased levels of depression in students [45]. A survey of students from public and private universities in Malaysia reported similar results [46]. The different results between the studies mentioned above and ours could be due to other diagnostic methods for depression and the diverse populations of students and university courses.

Likewise, each region’s economic, social, and personality factors can affect the prevalence of depression in students [47]. Latin American countries face aspects of their socioeconomic conditions that can affect mental health—for example, the lack of food in various areas of difficult access [48]. Alfayumi-Zeadna et al. reported that some economic and social factors that increased depression in Israeli students during the pandemic were: low income, job loss, region of residence, marital status, whether they own their home or not, and income level [49]. Yin et al. reported that medical students with low social support were more likely to have high levels of depression [50]. Browning et al. conducted a study in seven states in the United States where they analyzed the social and economic factors that affected students’ mental health during the COVID-19 pandemic. The main factors that influenced the students’ mental health were: not being in good health, spending little time outdoors, having low income, spending much time in front of the computer, and being a woman. The latter coincides with previous studies that have reported a higher prevalence of depression in women due to different factors such as hormones, interpersonal violence after childhood, body shame and dissatisfaction [51].

Gębska et al. analyzed the relationship between the appearance of physical symptoms (Stomatognathic System Disorders) and the stress generated during the COVID-19 pandemic in physiotherapy students. The authors found a connection between physical symptoms and students with type D personality (‘distressed personality’) [52]. Type D personality is a type of personality with the characteristic of being more susceptible and generating higher stress levels in complicated situations such as the COVID-19 pandemic. Due to the above, people with this personality type are also more vulnerable to developing moderate or severe levels of depression [53, 54]. With the presence of psychological disorders such as depression, not only did the frequency of temporomandibular disorders increase in students but also increased bruxism associated with depression in dental students during the pandemic [55]. Shailaja et al. reported that cyberchondria (when the excessive search for information about a disease on the internet increases the concern about the said disease) is also associated with high stress, anxiety, and depression levels in dental students during the COVID-19 pandemic [28].

Other studies have reported the co-occurrence of psychological disorders and alcohol abuse [56]. For example, the study by Fernandez et al. reported a relationship between alcohol abuse and moderate or severe anxiety levels in dental students in various regions of Brazil during the COVID-19 pandemic [57]. In addition, alcohol abuse by college students during the COVID-19 pandemic was associated with increased suicidal behavior [58–60]. The study by Chang et al. reported that students from rural areas and non-medical majors had fewer psychological symptoms (most had anxiety) compared with students from the suburbs and in medical majors (most had depression) [61]. As reported by Sanabria-Mazo et al., perhaps one of the main factors influencing the development of depression in Latin American students is social inequities (such as income level, employment status, education level, ethnic group, area of residence, and religion) [62]. Likewise, one way to reduce the psychological impact of COVID-19 on Latin American students is through self-employment and entrepreneurship, which helped reduce economic and social inequalities during the pandemic [63].

One of this study’s strengths is that the sample size was more extensive than most studies in similar populations. In addition, it was possible to analyze practically the entire population of interest in this study. Regarding the limitations, the questionnaires were applied individually, so we could not determine associations between the variables. The questionnaires were only used in one University, so it is difficult to extrapolate the results to the population of dental students throughout the country.

## Conclusions

According to the results of this study, 57% of the students presented moderate or severe levels of depression, which makes them candidates for receiving psychological attention. Therefore, this article contributes to a better understanding of this problem in this type of population [12]. Regardless of the levels of depression, the opinion of the students towards the hybrid learning model turns out to be quite good.

## Data Availability

The datasets used and analyzed during the current study are available from the corresponding author upon reasonable request.
